# Association between Childhood Maltreatment and Suicidal Ideation: A Path Analysis Study

**DOI:** 10.3390/jcm11082179

**Published:** 2022-04-13

**Authors:** Isabella Berardelli, Salvatore Sarubbi, Elena Rogante, Denise Erbuto, Carlotta Giuliani, Dorian A. Lamis, Marco Innamorati, Maurizio Pompili

**Affiliations:** 1 Department of Neurosciences, Mental Health and Sensory Organs, Suicide Prevention Centre, Sant’Andrea Hospital, Faculty of Medicine and Psychology, Sapienza University of Rome, Via di Grottarossa 1035, 00189 Rome, Italy; isabella.berardelli@uniroma1.it (I.B.); denise.erbuto@gmail.com (D.E.); 2 Department of Human Neurosciences, Sapienza University of Rome, Viale dell’Università 30, 00185 Rome, Italy; salvatore.sarubbi@uniroma1.it; 3 Department of Psychology, Sapienza University of Rome, Via dei Marsi 78, 00185 Rome, Italy; elena.rogante@uniroma1.it; 4 Psychiatry Residency Training Program, Psychiatry Unit, Sant’Andrea Hospital, Faculty of Medicine and Psychology, Sapienza University of Rome, 00185 Rome, Italy; karl_giuliani@alice.it; 5 Department of Psychiatry and Behavioral Sciences, Emory University School of Medicine, Atlanta, GA 30303, USA; dorian.lamis@emory.edu; 6 Department of Human Sciences, European University of Rome, Via degli Aldobrandeschi 190, 00163 Rome, Italy; marco.innamorati@unier.it

**Keywords:** suicide, suicidal ideation, childhood maltreatment, trauma, dissociative symptoms, hopelessness

## Abstract

Epidemiological studies have suggested that childhood maltreatment increases suicidal ideation, and dissociative symptoms and hopelessness are involved in this relation. To better address this issue, we used a path analysis model to examine the role of different types of childhood maltreatment on suicidal ideation, investigating whether hopelessness and dissociative symptoms mediated this relation. A sample of 215 adult psychiatric inpatients was enrolled between January 2019 and January 2020, at the psychiatric unit of Sant’Andrea Medical Center in Rome, Italy. The Childhood Trauma Questionnaire (CTQ), Beck Hopelessness Scale (BHS), Dissociative Experiences Scale (DES-II), and Columbia-Suicide Severity Rating Scale (C-SSRS) were used to test the hypotheses. Results revealed that the presence of sexual abuse directly affected suicidal ideation (β = 0.18, SE = 0.8, *p* < 0.05), while emotional abuse and neglect indirectly increased suicidal ideation via dissociation (β = 0.05, SE = 0.02, 95% C.I. 0.01/0.09) and hopelessness (β = 0.10, SE = 0.03, 95% C.I. = 0.04/0.16). Professionals working with children should be aware of the long-term consequences of childhood maltreatment, particularly suicide risk. Furthermore, professionals working with adults should inquire about past childhood maltreatment.

## 1. Introduction

Epidemiological studies have shown that childhood maltreatment, including physical, sexual, emotional abuse, and neglect, is present in about 30% of the population [1,2,3,4]. In addition, childhood maltreatment increases the risk of mental health consequences in adulthood, including depression, anxiety, psychosis, posttraumatic stress disorders, dissociative disorders, and personality disorders [5]. Risky sexual behavior and drug and/or alcohol addiction may also be present [6,7].

Several studies have also demonstrated an association between childhood maltreatment and suicidal ideation, defined as thoughts about a wish to be dead or active thoughts of wanting to end one’s life [8,9,10,11]. A recent meta-analysis of 68 studies showed that all types of childhood maltreatment were associated with significantly increased odds for suicidal ideation in adults. Notably, sexual abuse and emotional abuse were associated with a two-fold increased risk for suicidal ideation, physical abuse was associated with a 2.5-fold increased risk for suicidal ideation, and emotional and physical neglect was associated with a 1.5-fold increased risk for suicidal ideation [12]. A subsequent meta-analysis of 79 studies with 337,185 participants concluded that sexual abuse in young people was associated with 4.0-fold increased odds for suicide plans [13]. Several studies have also indicated that different types of childhood maltreatment predicted adult suicidal ideation [14,15,16]; Barbosa et al. [17], for example, found that childhood neglect, as well as physical and sexual abuse, were each associated with a threefold—and for emotional abuse a sixfold—increased risk of suicidal ideation in individuals aged 14–35 years. Moreover, childhood maltreatment is associated with suicide risk among patients with various psychiatric disorders. For example, Prokopez et al. [18] analyzed a sample of patients with schizophrenia and found that patients who experienced five or more adverse childhood experiences reported higher levels of suicidal ideation and more suicide attempts. An inclusive systematic review and meta-analysis [19] demonstrated that the presence of childhood maltreatments predicted a global clinical worsening and a higher suicidal risk in patients diagnosed with bipolar disorder. Furthermore, in a cross-sectional study on 473 patients with major depression, De Mattos Souza et al. [16] observed that emotional abuse and neglect were risk factors for suicidal ideation. Finally, in patients with psychotic disorders, childhood maltreatment was found to be associated with both clinical course and treatment outcomes [20].

Dissociative symptoms and hopelessness may play an important role among the various psychopathological features involved in the relation between childhood maltreatment and suicidal ideation. Dissociation is a complex psychopathological construct, defined as “disruption of and/or discontinuity in the normal integration of consciousness, memory, identity, emotion, perception, body representation, motor control, and behavior” [21]. The development of dissociative symptoms is considered a psychological adaptation strategy that allows a child to survive severe and prolonged abuse [22,23]. In contrast, hopelessness is a negative expectation toward oneself and the future [24]. According to Mueller-Pfeiffer et al. [25], childhood maltreatment is a strong predictor of dissociation.

Furthermore, Courtney et al. [26] demonstrated that childhood maltreatment predicted higher levels of hopelessness and depressive symptoms in adolescents. Indeed, hopelessness is one of the most prominent risk factors for suicide, as reported in several studies [27,28,29,30]. Finally, a recent meta-analysis [31] found that dissociative symptoms were strongly associated with suicidal risk; in an ideation-to-action framework to suicide, the progression from suicidal ideation to lethal suicidal attempts can be understood as a distinct process that includes dissociation as a factor that diminishes fear of pain, injury, and death and increases a person’s capability to attempt suicide [32].

Moreover, dissociative symptomatology has been proposed as one possible mediator in the link between childhood maltreatment and suicide risk, including suicide ideation and attempt [22,33,34,35,36,37,38]. Rodriguez-Srednicki [34] has analyzed the role of childhood sexual abuse on suicidal risk. Tamar-Gurol et al. [36] focused on the prevalence and correlates of dissociative disorders in a sample of drug addicts. Recently, Bertule et al. [38] conducted a study on patients with depression in a community sample, highlighting the complex interconnections between childhood abuse, dissociation, and suicidality. The authors demonstrated that emotional abuse was linked to dissociation, which, in turn, was related to depression and depression was linked to suicide ideation.

Previous studies have also suggested that hopelessness is related to childhood maltreatment [39,40]. Moreover, hopelessness is associated with suicide risk among psychiatric patients [41], particularly in those patients who have experienced childhood abuse and neglect [42]. Gibb et al. [43], inspired by the “hopelessness model” of suicide risk [44], hypothesized that childhood maltreatment might contribute to the development of a negative cognitive style that exacerbates hopelessness, which, in turn, triggers suicidal ideation and behaviors. Meadows and Kaslow [45] explored a sample of abused participants and found that hopelessness mediated the association between childhood maltreatment and suicide attempt.

Understanding the role of dissociative symptoms and hopelessness as possible mediators in the relation between the various types of childhood maltreatment and suicidal risk will advance our understanding of the association among these variables. To confirm and extend a previous path analysis study [38] that examined the associations between childhood trauma abuse, depressive symptoms, dissociative symptoms, and suicidality in patients with depression, the present study investigates the association between childhood maltreatment (both abuse and neglect) and suicidal ideation in patients with various psychiatric disorders. We hypothesized that different types of childhood maltreatment would be related to higher levels of hopelessness and dissociative symptoms, which would increase suicidal ideation in psychiatric patients. Moreover, dissociative symptoms and hopelessness would mediate the relationship between childhood maltreatment and suicidal ideation. Specifically, we have investigated the role of different types of childhood maltreatment (emotional neglect, emotional abuse, sexual abuse, and physical neglect and abuse) in suicidal ideation among patients with psychiatric disorders. Accordingly, we tested a path analysis model that belongs to a family of statistical techniques known as structural equation modeling (SEM), to assess the relationship between different types of childhood maltreatment, dissociative symptoms, hopelessness, and suicidal ideation.

## 2. Materials and Methods

### 2.1. Participants

This study was conducted on 215 adult psychiatric inpatients (46.5% women). The mean age of the patients was 39.72 years (standard deviation (SD) = 14.16 years; age range = 18–75 years). Patients were enrolled between January 2019 and January 2020 at the psychiatric ward of Sant’Andrea Medical Center, a teaching hospital of Sapienza University of Rome, Italy. Inclusion criteria were adult inpatients aged ≥ 18 years with a diagnosis of psychiatric disorder, according to DSM-5 [21]. Exclusion criteria were degenerative neurological diseases or comorbidities with alcohol or substance use disorders that prevented the patients from understanding and completing the questionnaires. All participants received a comprehensive explanation of the study procedures and goals, consistent with the Declaration of Helsinki. After signing a written informed consent form, all patients voluntarily participated in this study. The assessment of psychiatric patients with particular attention to suicide risk is part of several investigations approved by the local ethics review board.

### 2.2. Measures

Psychiatric diagnoses were made according to the Diagnostic and Statistical Manual of Mental Disorders, fifth edition (DSM-5) criteria [21] and supported by the Structured Clinical Interview for DSM-5 Disorders (SCID-5) [46]. A psychiatric diagnosis was made during the first days of hospitalization (48 h of the admission). Trained psychiatrists assessed suicide ideation when the patient arrived at the emergency department and admitted to the psychiatric ward. According to the definition adopted by Silverman for the assessment of suicide ideation, psychiatrists evaluated the presence and the degree of suicidal intent. Moreover, they investigated the presence of suicide attempts as self-inflicted, potentially injurious behaviors with a nonfatal outcome. There is explicit or implicit evidence of intent to die [47,48].

The Columbia-Suicide Severity Rating Scale (C-SSRS) [49,50] is a semi-structured interview used to evaluate suicide ideation and behaviors in individuals aged 12 years and older [50]. The C-SSRS consists of two sections, all of which maintain binary responses (yes/no) to indicate the presence or absence of the item. The first section evaluates the presence of suicidal ideation from the desire to be dead up to the presence of suicidal intentions with the planning of a specific method. The second section assesses suicide behaviors including actual, aborted, and interrupted attempts, preparatory and non-suicidal self-injurious behaviors and lethality of the attempts.

We only considered the first five items that investigate lifetime suicidal ideation for the study. The first two items assess the respondent’s wish to be dead (e.g., “Have you wished you were dead or wished you could go to sleep and not wake up?”) and nonspecific active suicidal thoughts (e.g., “Have you actually had any thoughts of killing yourself?”). If the patient replies affirmatively to either of these two items, the clinician asks three additional questions that assess active suicidal ideation with any method but with no plan or intent to act; active suicidal ideation with some intent to act but no plan; and active suicidal ideation with a specific plan and intent. The severity of suicidal ideation ranges from 0 (absence of suicidal ideation) to 5 (from 1 to 5, patients were considered at some degree of risk for suicide), based on the maximum suicidal category observed (the higher item with a positive answer). This section showed excellent reliability (α = 0.916).

The Childhood Trauma Questionnaire (CTQ) [51,52] is a 28-item self-report questionnaire to assess emotional and physical abuse, sexual abuse, and emotional and physical neglect. Each item begins with the anchor, “when I was growing up” and respondents indicate the frequency of a particular incident on a 5-point Likert scale (1 = never true; 5 = very often true). Each 5-item subscale ranges from 5 (no history of abuse) to 25 (very extreme history of abuse). Each scale has a different cut-off score and can detect four levels of maltreatment (none or minimal, low to moderate, moderate to severe, severe to extreme); in our study, mean scores for each subscale indicated a low to moderate level of maltreatment [52]. Subscale reliability in this study were emotional neglect α = 0.771, emotional abuse α = 0.776, sexual abuse α = 0.895, physical neglect α = 0.402, and physical abuse α = 0.795.

The Beck Hopelessness Scale (BHS) [53,54] is a 20-item self-report scale that measures negative attitudes about the future (e.g., “I look forward to the future with hope and enthusiasm”, “I might as well give up because I can’t make things better for myself”). This powerful predictor of eventual suicide addresses three major aspects of hopelessness: feelings about the future, loss of motivation, and expectations. In Italy, validation studies have been conducted on samples of medical patients, university students, and psychiatric inpatients and have demonstrated satisfactory psychometric properties [55]. Several studies indicated that, in psychiatric samples, the BHS is a valid measure for predicting subsequent suicide behavior [56,57]. For example, a score of ≥9 is able to detect patients at risk for suicide [57,58]. In the current study, the scale had good reliability (α = 0.890).

The Dissociative Experiences Scale (DES) [59] is a self-rating instrument comprising 28 items that build on the assumption of a “dissociative continuum” ranging from mild normative to severe pathological dissociation. Patients were asked to circle the percentage of time in which they had the experience described, including questions on experiences of amnesia, absorption, depersonalization, and derealization (e.g., “Some people have the experience of driving a car and suddenly realizing that they don’t remember what has happened during all or part of the trip”). A score ≥ 30 can be used to distinguish those patients with mild and severe levels of dissociation [60]. In the present study, we used the Dissociative Experiences Scale–II (DES-II) [59], a revised version of the scale, developed because the former version’s scoring procedure was time-consuming. DES-II uses an 11-point Likert scale ranging from 0 to 100, demonstrating excellent reliability (α = 0.945). In this study, we administered the Italian version of the scale [61]

### 2.3. Statistical Analysis

Descriptive analysis was performed with the Statistical Package for Social Sciences (IBM SPSS Statistics for Mac, v.25, Armonk, NY, USA) and reported with means, standard deviations (e.g., age), and percentages (e.g., sex, job, diagnosis). To test the study hypotheses, a path analysis model was performed with Mplus v.8 software (Muthén & Muthén, Los Angeles, CA, USA) [62]. Unweighted least square (ULS) estimator was used to control for the non-normal variable distribution after evaluating the study’s variables for skewness and kurtosis. The model included pathways through which childhood maltreatment dimensions, hopelessness, and dissociation contribute to suicidal ideation in a sample of psychiatric inpatients with various diagnoses. Suicidal ideation was considered as a categorical variable. The goodness of fit was examined using standard fit statistics such as chi-square statistics, the Tucker–Lewis index (TLI), the root mean square error of approximation (RMSEA), and the standardized root mean square residual (SRMR). Acceptable model fit was reached with a non-significant chi-square test, TLI and CFI values of 0.90 or above, RMSEA values < 0.080, and SRMR values below 0.060 [63]. Indirect effect estimates were tested through empirical sampling distributions by calculating confidence limits and testing statistical significance of indirect effects through 0 within the interval. All path coefficients were reported as standardized estimates. A path diagram representing the model was constructed, with straight arrows representing significant regression paths, dashed arrows representing non-significant regression paths, and curved arrows representing correlations. All tests were considered significant with a *p* value < 0.05.

## 3. Results

### 3.1. Descriptive Analysis

The sociodemographic and clinical characteristics of the sample are reported in Table 1. Most patients were male (53.5%), single (57.2%), employed (50.2%), and well educated (54%). The mean age of the sample was 39.7 (SD = 14.1, age-range = 18/75 years). Regarding psychiatric diagnoses, 36.2% of patients had a diagnosis of schizophrenia or other psychoses, 38.1% had mood disorders (25.1% bipolar disorders; 13% major depression), and 10.7% had other psychiatric disorders. Concerning lifetime suicidal ideation, 23.7% of the patients referred absence of suicidal ideation, 7.4% referred a wish to be dead, 10.3% referred suicidal thoughts, 18.6% referred suicidal intent, and 40% of the patients referred suicidal intent with a specific plan. Moreover, 54.9% of patients referred at least one lifetime suicide attempt. Regarding the type of childhood maltreatment, 53% of patients referred to the presence of emotional abuse, 29.8% referred to physical abuse, 37.8% sexual abuse, 67.9% emotional neglect, and 50.2% of the patients referred to physical neglect.

### 3.2. Path Analysis

**Figure 1:**
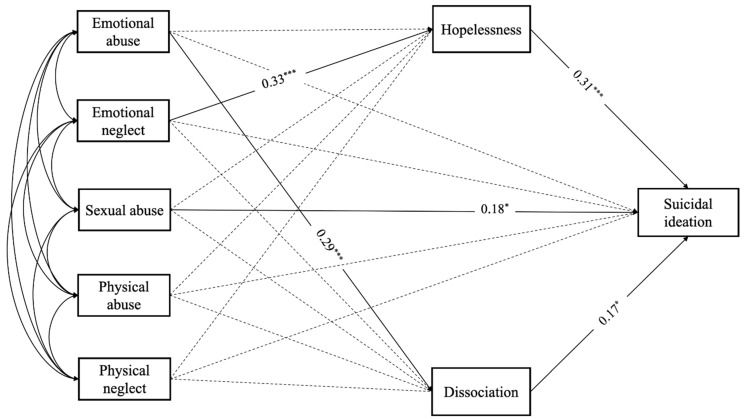
Path analysis. All reported parameters are standardized. * *p* < 0.05; *** *p* < 0.001. For ease of interpretation, the effects of covariates are not shown in the figure.

Path analysis is shown in Figure 1. The model fit information follows: χ^2^_1_ = 0.277, *p* = 0.598; CFI = 1.0; RMSEA (90% C.I. = 0.00 (0.00–0.15)); SRMR = 0.006. Overall, the R-square index showed that the childhood trauma dimensions, hopelessness, and dissociation were able to explain 22% of the total variance in the suicidal ideation (R^2^ = 0.218, *p* < 0.001).

Results for emotional abuse indicated a significant total effect on suicidal ideation (β = 0.19, *p* < 0.05). The total indirect effect was significant (β = 0.10, SE = 0.03, 95% C.I. = 0.04/0.15); in particular, emotional abuse had an indirect effect on suicidal ideation via dissociation (β = 0.05, SE = 0.02, 95% C.I. 0.01/0.09). Specifically, emotional abuse had a positive and significant effect on levels of dissociation (β = 0.29, *p* < 0.001); in turn, dissociation had a positive effect on suicidal ideation (β = 0.17, *p* < 0.05). 

Emotional neglect did not have a significant total effect on suicidal ideation; however, the total indirect effect was significant (β = 0.10, SE = 0.04, 95% C.I. = 0.03/0.16) and, in particular, emotional neglect had an indirect effect on suicidal ideation via hopelessness (β = 0.10, SE = 0.03, 95% C.I. = 0.04/0.16). Specifically, emotional neglect had a significant effect on levels of hopelessness (β = 0.33, *p* < 0.001) and, in turn, hopelessness had a positive effect on suicidal ideation (β = 0.31, *p* < 0.001).

Sexual abuse was found to have a significant total effect on suicidal ideation (β = 0.20, *p* < 0.05); in particular, sexual abuse had a positive and direct effect on suicidal ideation (β = 0.18, SE = 0.8, *p* < 0.05), while no indirect effects were significant.

Lastly, neither physical abuse nor physical neglect showed significant effects on suicidal ideation.

## 4. Discussion

The present study results have shown that emotional abuse was related to suicidal ideation through the mediating role of dissociation. Furthermore, emotional neglect was related to suicidal ideation through the mediating role of hopelessness. Finally, sexual abuse directly affected suicidal ideation, whereas physical abuse and neglect were not associated directly or indirectly—through the mediators—with suicidal ideation.

Regarding emotional abuse, we found a relation to suicidal ideation through the mediating role of dissociation. These results confirm previous studies that demonstrated the role of emotional maltreatment on psychopathological consequences [64,65,66,67,68]. Recently, Bertule et al. [38] conducted a path analysis highlighting the complex interconnections between childhood abuse (emotional, physical, and sexual), dissociation, and suicidality among patients with depression in a community sample. Specifically, emotional abuse was linked to dissociation, which, in turn, was related to depression and ultimately predictive of suicide ideation. However, this study only included patients with a single psychiatric diagnosis.

Furthermore, the results indicated that emotional neglect was associated with suicidal ideation through the mediating role of hopelessness. Interestingly, the results suggested two different pathways for emotional abuse and neglect. In particular, emotional abuse was linked to suicidal ideation through dissociation, while emotional neglect through hopelessness. Consistent with the hopelessness theory [69], we hypothesized that emotionally neglected children develop a negative perception of themselves, of others, and of the future (hopelessness), increasing suicide risk. Otherwise, people who experienced emotional abuse may develop dissociative symptoms [70]. These findings can be partially explained because childhood emotional abuse is often inflicted by somebody close to the victim, producing dissociative experiences that protect the child’s internal self [70]. Moreover, our results have shown that sexual abuse directly affected suicidal ideation [71,72,73]. Childhood sexual abuse has serious, long-term medical and psychological consequences [74,75], and produces a cascade of neurobiological events linked also to suicidal ideation [76]. 

Finally, through the mediators, physical abuse and neglect were not associated directly or indirectly with suicidal ideation. This result is consistent with the findings of Gibb and colleagues [43], who demonstrated that only emotional maltreatment was associated with suicidal ideation across a 2.5-year follow-up. However, it is in contrast with many other studies that have highlighted the contributory role of physical maltreatment on suicidal ideation and behavior [12,77]. Hence, it is important to conduct further studies to investigate the association between these variables more thoroughly.

Assessing the relationship between different types of childhood maltreatment and suicide risk in psychiatric patients is fundamental for a more appropriate multidisciplinary management of these patients. Several studies suggested a neuropsychological relation between childhood maltreatment and suicide risk in psychiatric patients. Savitz et al. [78] reported an association between emotional, physical, and sexual abuse with poorer cognitive performances in patients with bipolar spectrum disorders. McLaughlin et al. [79] proposed a transdiagnostic model to explain better the association between childhood trauma and psychopathology, based on elevated emotional reactivity to threat-related stimuli, low emotional awareness, and difficulties with emotional learning and emotion regulation. Furthermore, childhood maltreatment has been shown to contribute to many structural and functional changes in the brain, including such structures as the hippocampus and amygdala, associated with several psychiatric disorders and suicide risk [80].

Our study has several limitations. First, temporal ordering between the predictor and outcome of interest (i.e., childhood sexual abuse occurring before 18 years of age and outcome measurement occurring after 18) was not part of the inclusion criteria. This feature may have increased the possibility of conflating childhood abuse’s short-term effects on long-term adult consequences. Second, we did not investigate maltreatment duration nor the age at which it occurred, and no information was collected regarding who inflicted the maltreatment. Third, our sample may not be sufficiently representative. Moreover, psychiatric conditions may affect suicidal risk, regardless of the role of childhood maltreatment, hopelessness, and dissociation, limiting the study results’ generalizability to the general population. Fourth, the cross-sectional design retrospectively assessed self-report data and, thus, possible memory bias regarding child maltreatment should be considered when interpreting our results. Furthermore, patients tend to under-report abuse and suicide-related constructs, so social-desirability bias should be considered.

## 5. Conclusions

The present study results demonstrate that different types of childhood maltreatment increase suicidal risk in adult psychiatric patients and that hopelessness and dissociation are important mediators of this association. Moreover, an interesting result is represented by the different paths of emotional abuse and emotional neglect on suicidal ideation, suggesting the importance of conducting additional studies. In addition to psychiatric diagnoses, many psychological factors play a role in suicide. Professionals working with children should be aware of the long-term consequences of childhood maltreatment, particularly suicidal ideation. Consequently, it is important to identify factors that promote resiliency among individuals who have experienced maltreatment and protect them against increased suicide risk.

Furthermore, our results emphasize the importance for professionals working with adults to inquire about past childhood maltreatment, given the relationship between childhood maltreatment, poor clinical course, and treatment response in several psychiatric populations. Therefore, clinicians should consider the paths to suicide risk and implement interventions that take specific risk factors, such as childhood maltreatment, dissociation, and hopelessness, into account. Future studies are needed to verify and explain the relationship between childhood maltreatment and mental disorders. These studies should utilize larger prospective samples and instruments to complement self-rating scales and control for additional demographic features.

## Figures and Tables

**Table 1:** Sociodemographic and clinical characteristics of the sample (N = 215).

Variables	N	%
Sex		
Male	115	53.5
Female	100	46.5
Age—M ± SD	39.72 ± 14.1	
Marital status		
Married	65	30.2
Divorced or widowed	27	12.6
Single	123	57.2
Job		
Employed	108	50.2
Unemployed	93	43.3
Other	14	6.5
Years of education		
<8 years	62	28.8
9–15 years	116	54.0
>16 years	37	17.2
Diagnosis		
Schizophrenia and other psychoses	78	36.2
Bipolar disorders	54	25.1
Major depression	28	13.0
Other	23	10.7
Comorbidity		
Personality disorder	27	12.6
Addiction	5	2.3
Severity of suicidal ideation M ± SD	3.08 ± 2.1	
Lifetime suicide attempt	118	54.9
Emotional abuse M ± SD	9.69 ± 4.6	
Emotional neglect M ± SD	12.29 ± 5.1	
Sexual abuse M ± SD	7.24 ± 4.3	
Physical abuse M ± SD	7.19 ± 3.5	
Physical neglect M ± SD	7.80 ± 3.0	
BHS M ± SD	8.55 ± 5.4	
DES-II M ± SD	24.4 ± 17.8	

## Data Availability

Data available on request due to restrictions, e.g., privacy or ethical. The data presented in this study are available on request from the corresponding author.
